# The Kansas Medical Journal

**Published:** 1889-06

**Authors:** 


THE KANSAS MEDICAL JOURNAL, published monthly at Topeka, made its first appearance in May. It is edited by a committee composed as follows: W. L. Schenck, M. D., J. E. Minney, M. D., and S. G. Stewart, M. D. It is a 32-page quarto, and presents an unusually good appearance for a first number. We extend a cordial greeting to our new contemporary.

